# DDX5 Facilitates HIV-1 Replication as a Cellular Co-Factor of Rev

**DOI:** 10.1371/journal.pone.0065040

**Published:** 2013-05-31

**Authors:** Xiuxia Zhou, Juan Luo, Lisa Mills, Shuangxin Wu, Ting Pan, Guannan Geng, Jim Zhang, Haihua Luo, Chao Liu, Hui Zhang

**Affiliations:** 1 Institute of Human Virology, Zhongshan School of Medicine, Sun Yat-sen University, Guangzhou, China; 2 Key Laboratory of Tropical Disease Control of Ministry of Education, Zhongshan School of Medicine, Sun Yat-sen University, Guangzhou, China; 3 Cyrus Tang Hematology Center, Jiangsu Institute of Hematology, First Affiliated Hospital, Soochow University, Suzhou, China; 4 Center for Human Virology, Division of Infectious Diseases, Department of Medicine, Thomas Jefferson University, Philadelphia, Pennsylvania, United States of America; Institut Pasteur, France

## Abstract

HIV-1 Rev plays an important role in the late phase of HIV-1 replication, which facilitates export of unspliced viral mRNAs from the nucleus to cytoplasm in infected cells. Recent studies have shown that DDX1 and DDX3 are co-factors of Rev for the export of HIV-1 transcripts. In this report, we have demonstrated that DDX5 (p68), which is a multifunctional DEAD-box RNA helicase, functions as a new cellular co-factor of HIV-1 Rev. We found that DDX5 affects Rev function through the Rev-RRE axis and subsequently enhances HIV-1 replication. Confocal microscopy and co-immunoprecipitation analysis indicated that DDX5 binds to Rev and this interaction is largely dependent on RNA. If the DEAD-box motif of DDX5 is mutated, DDX5 loses almost all of its ability to bind to Rev, indicating that the DEAD-box motif of DDX5 is required for the interaction between DDX5 and Rev. Our data indicate that interference of DDX5-Rev interaction could reduce HIV-1 replication and potentially provide a new molecular target for anti-HIV-1 therapeutics.

## Introduction

The Rev protein of human immunodeficiency virus type 1 (HIV-1) is a 19 kDa protein produced from fully spliced mRNA in the early phase of HIV-1 gene expression, and functions as a nucleocytoplasmic shuttling phosphoprotein [1]. Rev is a key regulator of HIV-1 replication because it enables the transition from the early phase of gene expression to the late phase [2], [3]. Binding to unspliced and incompletely spliced HIV-1 transcripts and shuttling of these mRNAs from the nucleus to cytoplasm are the best-characterized function of Rev [4]. The efficient export of nuclear/cytoplasmic RNA is accomplished by binding to the Rev Response Element (RRE) within these mRNAs [5]. The RRE sequence spans approximately 350 nucleotides (nt), is located within the *env* region of unspliced or incompletely spliced mRNAs, and is absent in completely spliced mRNAs [6], [7]. In addition to the export of unspliced or incompletely spliced mRNA, Rev also enhances their translation and increases the half-life of RRE-containing mRNAs in the nucleus [3].

Many Rev co-factors have been identified, including CRM1 (chromosome maintenance region 1) and several members of the DEAD-box RNA helicase family [8], [9], [10]. The DEAD-box protein family is a group of RNA helicases that play roles in many biological processes such as transcription, pre-mRNA splicing and export, ribosomal biogenesis, translational initiation, and RNA decay [11], [12], [13]. The motif for which these proteins are named contains the highly conserved Asp-Glu-Ala-Asp amino acid sequence, which is known as the DEAD-box. This motif (described as motif II), together with motif I, Q-motif, and motif VI, is required for ATP binding and hydrolysis. In addition, DEAD-box helicases carry out their functions with some co-factors that increase helicase specificity and enzymatic activity [14], [15], [16].

In recent years, genome-wide screening technologies have been used by several groups to clarify cellular factors that affect HIV-1 replication. More than 300 cellular factors have been identified as a result of these studies [5], [17], [18], [19], [20], [21], [22]. Among these factors, several are members of the DEAD-box helicase family including DDX5 (P68) [18]. DDX5 is a multifunctional DEAD-box RNA helicase. It functions as an enzyme that unwinds double-stranded RNA and is a nucleocytoplasmic shuttling protein whose action is mediated by a Ran GTPase-dependent pathway [23], [24]. Previously studies identified DDX1 and DDX3 as co-factors of Rev in the export of unspliced and partially spliced HIV-1 mRNAs from the nucleus to cytoplasm [9], [10]. Like its counterparts, DDX5 may also be involved in the Rev/RRE-dependent pathway of HIV-1. Through various approaches, we herein demonstrated that DDX5 functions as a new co-factor of HIV-1 Rev and that it enhances the transport of HIV-1 transcripts.

## Materials and Methods

### Ethics Statement

This research was approved by the Ethics Review Board of Sun Yat-Sen University. The written informed consent was provided by study participants and/or their legal guardians.

### Plasmids

Human *DDX5* with an HA or FLAG epitope tag sequence at its 3′ terminus was amplified through reverse transcription-polymerase chain reaction (RT-PCR) with the mRNA of human peripheral blood mononuclear cells (PBMCs) as template. Accuracy was confirmed by DNA sequencing. The tagged *DDX5* was then inserted into a pcDNA3.1 vector. pcDNA3.1-Rev, expressing HIV-1 Rev, was constructed as previously described [25]. The *gfp* and HIV-1 *rev* with HA tag sequences at their C-termini were amplified from pEGFP-C1 (Clontech) or pcDNA3.1-Rev via PCR, and the accuracy was confirmed by DNA sequencing. The tagged *gfp* or *rev* was then inserted into a pcDNA3.1 vector. HIV-1 *rev* and human *DDX5* were PCR amplified from pcDNA3.1-Rev or pcDNA3.1-DDX5-HA, and then *rev* was inserted into pEGFP-N1 (Clontech) to generate pEGFP-N1-Rev, respectively. The DDX5-DEAD-box mutant plasmid pcDNA3.1-DDX5 mutant-FLAG and pcDNA3.1-DDX5 mutant-HA were constructed via PCR-based mutagenesis from pcDNA3.1-DDX5-FLAG or pcDNA3.1-DDX5-HA by replacing the DEAD-box motif with 4 alanine amino acids. pDM628, a Rev/RRE-dependent reporter vector, was constructed as previously described [9], [26]. pRL-TK, which expresses *renilla* luciferase, was obtained from Promega as a transfection normalization reporter. pNL4-3 contains a full infectious clone of HIV-1 provirus [27]. HIV-1 provirus pNL4-3-ΔEnv-GFP was constructed by Dr. Siliciano′s lab and obtained from AIDS Reference Reagent Program of NIH [28]. The HIV-1-based second generation packaging vector pCMVΔR8.2 (lacking only the *env* gene) was directly obtained from Dr. Trono′s lab [29]. pMDLg/RRE is a third generation lentiviral packaging plasmid that contains HIV-1 *gag* and *pol* genes as well as the element encoding HIV-1 RRE, and was obtained from Dr. Trono′s lab via Addgene. To replace the RRE sequence with a Constitutive Transport Element (CTE) of the type D Mason-Pfizer monkey virus (MPMV), two rounds of PCR amplification were performed as follows. In the first round of PCR, three fragments were amplified. The 191-bp CTE-containing fragment was derived from pDM128/CTE (CTE-up: 5'-ATTCCGGAGCGGCCGCAAGACTGGACAGCCAATGACGGGTA; CTE-dn: 5'-CCAGAGCAACCCCAAATCCCCCACATCCCTCGGAGGCTGCGCCTG-3') [8]. A 393-bp fragment was PCR amplified from pMDLg/RRE using primers 393-up: 5'-CCGCTTAAGACAGCAGTACAAATGGCAGT-3' (the *Afl*II site is underlined) and 393-dn: 5'-TGGCTGTCCAGTCTTGCGGCCGCTCCGGAATTCCATGTGT-3'. The third fragment (166 bp) was also derived from pMDLg/RRE via PCR (166-up: 5'-GCCTCCGAGGGATGTGGGGGATTTGGGGTTGCTCTGGA-3'; 166-dn: 5'-TCCCCGCGGAAGCTTGTGTAATTGTTA, the *Sac*II site is underlined). In the second round of PCR, the three PCR products above were mixed in an equimolar ratio and amplified with 393-up primer and 166-dn primer. The 682-bp *Afl*II*-Sac*II-flanked fragment, containing the CTE sequence, was digested with *Afl*II and *Sac*II, and cloned into the pMDLg/RRE vector that was digested with the identical enzymes so as to construct the recombinant plasmid pMDLg/CTE.

### Cells and Transfection

TZM-bl cells, which harbor an HIV-1 promoter-driven luciferease gene, were obtained from AIDS Reference Reagent Program, NIH. Human 293T, HeLa, and TZM-bl cells were maintained in Dulbecco′s modified Eagle′s medium (DMEM) (Hyclone) supplemented with 10% fetal bovine serum (FBS) (Invitrogen), 100 units/ml of penicillin and 100 μg/ml of streptomycin at 37°C. The 293T, HeLa, and TZM-bl cells were transfected using Lipofectamine 2000 (Invitrogen) for plasmids and siRNAs. The procedures recommended by the manufacturer were followed. The cells were collected at 48 h post-transfection (p.t.) for reporter and protein expression assays.

### siRNA Synthesis

Small interfering RNAs (siRNAs) for human *DDX5* and *gfp* were purchased from Dharmacon. The target sequence in human *DDX5* for siRNA was 5′-CCGCAACCAUUGACGCCAUTT-3′ [30]. The target sequence in *gfp* for siRNA was 5′-ACGTAAACGGCCACAAGTTC-3′. siRNA for *gfp* was used as a negative control.

### Luciferase Assay

The luciferase assay was performed as described previously [31], [32].

### Purification and Activation of Human Primary CD4+T Cells

Peripheral blood mononuclear cells (PBMCs) were isolated from normal human donors through Ficoll gradient centrifugation, followed by culturing in conditioned RPMI 1640 medium. Human primary CD4+T cells were then purified with human CD4+T cell isolation kit according to the manufacturer's instructions (Miltenyi Biotec).

Human primary CD4+T cells were stimulated with phytohemagglutinin (PHA, 5 ng/ml) and interleukin-2 (IL-2, 10 ng/ml) for 48 h, and then, cells were washed three times with phosphate-buffered saline (PBS) buffer, and cultured in the presence of IL-2 (10 ng/ml). Every three days, the culture was added with half volume of fresh conditioned RPMI 1640 medium containing IL-2 (10 ng/ml).

### Production of HIV-1

293T cells were transfected with 10 μg of pNL4-3 by using Lipofectamine 2000 (Invitrogen) according to manufacturer's instructions. Cells supernatants were harvested at 48 h post-transfection (p.t.) and stored at −80°C.

### p24 ELISA Assay for Plasmids Transfection and HIV-1 Infection

293T cells were seeded in 24-well plates (0.5×10^5^ cells/well) and transfected with target plasmids. Viral supernatants were collected at 48 h p.t.

To normalize viral inputs, the amount of p24 was measured by HIV-1 p24 ELISA kit according to manufacturer's instructions (Clonetech). Human primary activated CD4+T cells were seeded into 24-well cell culture plates (1×10^6^ cells/well) and infected with the equivalent of 5 ng HIV-1 p24 antigen in 1 ml for 3 h at 37°C. And then, supernatants were removed and cells were washed three times with fresh PBS buffer. The cells were maintained in conditioned RPMI 1640 medium supplemented with IL-2 (10 ng/ml) and transfected with 30 nmol DDX5-siRNA (GFP-siRNA as a control) every 2 days by using RNAiMAX (Invitrogen). Viral supernatants were harvested after 5 days post-infection.

All of the viral supernatants were detected using a HIV-1 p24 ELISA kit according to the manufacturer's instructions (Clonetech).

### Preparation of HIV-1 p24 Antibody

HIV-1 p24 expression frame, which was amplified from pNL4-3 via PCR, was inserted into pET28a, a prokaryotic expression vector (Novagen). Then, HIV-1 p24 was expressed in *E. coli* with a His-tag at the 5′-terminus. The protein was then purified through the immobilized metal ion affinity chromatography (IMAC). The purity of isolated recombinant protein was approximately 95%, as measured by Coomassie brilliant blue staining. Two NZW SPF rabbits (New Zealand white rabbits that are specific pathogen-free) were used for immunization. The subcutaneous injections were completed as the emulsions mixed with complete Freund's adjuvant (CFA) or incomplete Freund's adjuvant (IFA). After immunonization four times, cardiac blood samples were collected. The IgG was isolated from the rabbit serum through protein A (GeneScript L00210) affinity purification. The efficacy of the antibody was measured by ELISA and Western blotting.

### Co-immunoprecipitation and Western Blotting

In preparation for transfection, 1.5×10^6^ HeLa cells were plated onto 60-millimeter (mm)-diameter cell culture plate and grown at 37°C in 5 ml Dulbecco's modified Eagle's medium (DMEM). The cells were then transfected with 6 μg pcDNA3.1-DDX5-HA or pcDNA3.1-GFP-HA. After 24 h, cells were collected and treated with lysis buffer [150 mM NaCl, 50 mM Tris-HCl (pH 7.5), and 1mM EDTA, 1% Triton X-100, and 0.5% NP-40]. Co-immunoprecipitation and Western blotting were then performed as previously described [32]. The anti-HA antibody (mouse monoclonal, Covance), anti-β-actin antibody (rabbit polyclonal, CST), and anti-p24 antibody were used as primary antibodies. Quantity One (Biorad) was used to quantify the Western blotting results.

### Immunofluorescence and Confocal Imaging

HeLa cells were seeded onto 35-mm glass-bottom culture dishes (MatTek) and then co-transfected with 200 ng pEGFP-N1-Rev and 800 ng pcDNA3.1-DDX5-HA (or pcDNA3.1-DDX5-mutant-HA). At 36 h p.t., the dishes were washed with PBS buffer and the cells were fixed with 4% paraformaldehyde for 10 min at room temperature (RT). The dishes were then washed by PBS buffer for three times and were immersed in 0.2% Triton X-100 solution for 10 min at RT, followed by washing with PBS buffer for three times. The dishes were treated with 5% BSA blocking solution for 30 min and then washed by PBS buffer for two times. The anti-HA antibody solution was added for 1 h at RT and then washed by PBST buffer for three times. The secondary antibody (Goat anti-mouse IgG, purchased from Abcam) was subsequently added and incubated for 45 min at RT, followed by washing for four times. DAPI solution (0.5 μg/ml) was then added and subsequently washed by PBS buffer for three times. The cells on the dishes were examined with a Leica laser-scanning fluorescence microscope. All images were digitally recorded and merged using the Leica software. The magnification used to collect images was 600.

### Real-time RT-PCR

Primers *firefly luciferase*-F (5′-TGGGCGCGTTATTTATCGGA-3′) and *firefly luciferase*-R (5′-CACTACGGTAGGCTGCGAAA-3′) were synthesized for detection of the *firefly luciferase* gene in pDM628. Primers *renilla luciferase*-F (5′-GCCAGTAGCGCGGTGTATTA-3′) and *renilla luciferase*-R (5′-AAATGCCAAACAAGCACCCC-3′) were synthesized for detection of the *renilla luciferase* gene in pRL-TK. Two pairs of RT-PCR primers, 628-F (5′-GAAGAAGCGGAGACAGCGACGAAGAGCTC-3′)/628-dn-RT (5′-CCAGCGGTTCCATCCTCTAGAGGATAGA-3′) and 628-F/628-Sp-RT (5′-CTAAAGCTGCCTTGTAAGTCATTGGTC-3′) were synthesized by Invitrogen to detect unspliced and spliced mRNAs generated from pDM628. Primers *gag*-*pol*-F and *gag*-*pol*-R were used for detection of *gag*-*pol* mRNA (*gag*-*pol*-F, 5′-CGATAGACAAGGAACTGTA-3′; *gag*-*pol*-R, 5′-TGACAGGTGTAGGTCCTACT-3′). Primers *gag*-F (5′-TGGCTTCTTATGCGACGGATCG-3′) and *gag*-R (5′-CTCCCTGCTTGCCCATACTA-3′) were synthesized for detection of the *gag* gene. Primers *tat*-F and *tat*-R were used for detection of the *tat* gene (*tat*-F, 5′-GAGCCAGTAGATCCTAGACTAGA-3′; *tat*-R, 5′-CCCTCGGGATTGGGAGGTG-3′). Primers *rev*-F and *rev*-R were used for detection of the *rev* gene (*rev*-F, 5′-AGACTCATCAAGCTTCTCTATC-3′; *rev*-R, 5′-TTCCACAATCCTCGTTACAATC-3′). Fractionation of cytoplasmic and nuclear components and RNA extraction were performed according to the manufacturer's instructions (PARIS, Ambion). Reverse transcription reactions were performed with PrimeScript RT reagent Kit (TaKaRa). Real-time PCR procedures recommended by the manufacturer were followed (SYBR Premix ExTaq, TaKaRa) on a CFX96 Real-Time System (Bio-Rad). Human GAPDH and/or β-actin mRNA was measured as an endogenous control.

## Results

### Importance of DDX5 for HIV-1 Replication

**Figure 1:**
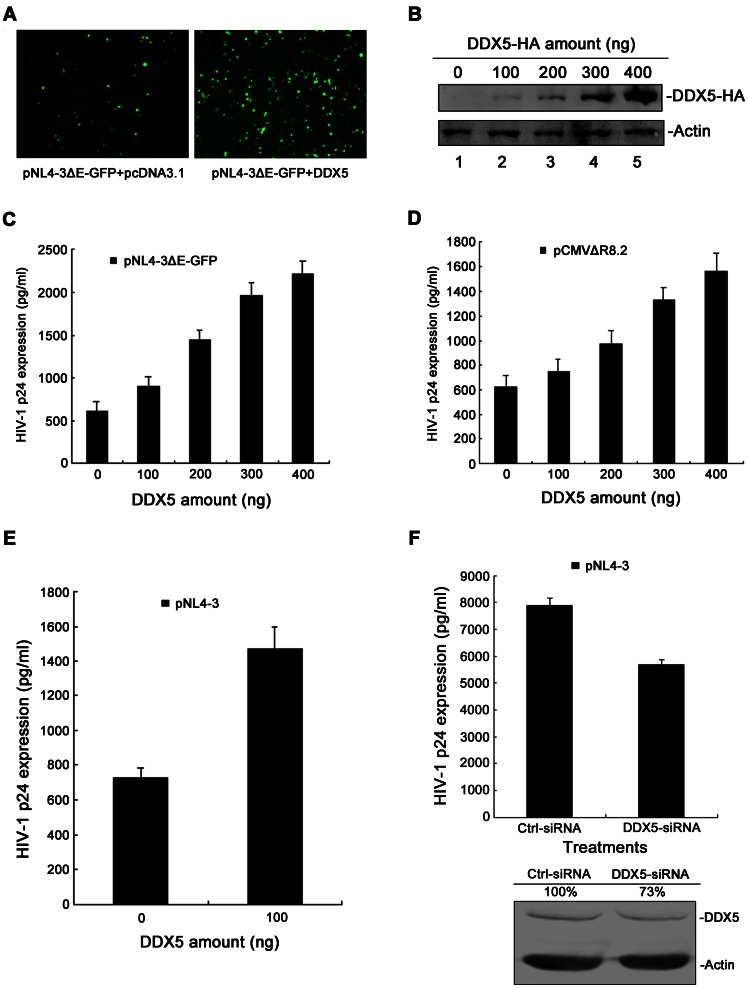
DDX5 is important for HIV-1 replication. **A**. Fluorescence images show 293T cells co-transfected with pNL4-3ΔEnv-GFP and pcDNA3.1-DDX5-HA (or pcDNA3.1) at 48 h p.t. **B**. Western blotting analysis of DDX5 expression in 293T cells. **C**, **D** and **E**. HIV-1 p24 ELISA assay of 293T cells supernatants. 293T cells in 24-well plates were co-transfected with pNL4-3ΔEnv-GFP (or pCMVΔR8.2, pNL4-3) and differing amounts of pcDNA3.1-DDX5-HA (pcDNA3.1 as a control). The supernatants were collected at 48 h p.t. for assay of p24 ELISA. **F**. The effect of DDX5 knockdown on HIV-1 p24 production. Top, the supernatants from human primary CD4+T cells infected with pNL4-3 in the presence of DDX5-siRNA (GFP-siRNA as a control) were collected at 5 days post-infection and analyzed with p24 ELISA kit. Bottom, the effect of DDX5-siRNA in human primary CD4+T cells was detected by Western blotting. Data in **C**, **D**, **E** and **F** represent mean ±S.D. (error bars).

Recently, Bushman et al analyzed the full panel of HIV-1 infection-associated proteins [18]. Among these proteins, DDX5 is involved in Rev-associated complex, indicating its specific links to HIV-1 replication [18]. Previous studies have found that some DEAD-box RNA helicases, such as DDX1, DDX3, DDX10, DDX53 and DDX55, are required for HIV-1 replication [9], [10], [17]. As DDX5 also belongs to the DEAD-box RNA helicase family, we hypothesized that DDX5 could function as a co-factor in HIV-1 replication. To this end, we transfected 293T cells with the HIV-1 molecular clone pNL4-3ΔEnv-GFP and the DDX5-HA-expressing plasmid. The transfected cells could then be monitored by the fluorescence of GFP. Compared to negative control, a significant difference was observed in DDX5-expressing cells at 48 h post-transfection (p.t.) (Fig. 1A). To further confirm this finding, a dose-dependent experiment was performed. Firstly, we transfected 293T cells with differing amounts of pcDNA3.1-DDX5-HA and detected the expression of DDX5 in cells by Western blotting. Then, the 293T cells were transfected with pNL4-3ΔEnv-GFP and differing amounts of DDX5-HA-expressing plasmids. Forty-eight hours later, p24 expression of cell culture supernatants was analyzed. Compared to the vector control, DDX5 enhanced the production of HIV-1 p24 significantly and this increase correlated with the expression level of DDX5 (Fig. 1C). The same phenotype was recapitulated with two other types of HIV-1 molecular clones (pCMVΔR8.2 and pNL4-3; Fig. 1D and E). During DDX5 overexpression, the 0 ng DDX5 transfection lanes in Fig. 1C–E reveal basal levels of p24 due to the expression of endogenous DDX5. To better define the effect of DDX5 on HIV-1 replication, a DDX5-knockdown experiment was performed. Human primary CD4+T cells were infected with HIV-1_NL4-3_ viruses in the presence of DDX5-siRNA. Five days later, HIV-1 p24 expression of cell culture supernatants was analyzed. After depletion of endogenous DDX5, p24 production from wild-type HIV-1_NL4-3_ infection was reduced by 30% (Fig. 1F, top panel) and the p24 production of another type of HIV-1 clone (pNL4-3ΔEnv-GFP) was decreased by ∼72% (Fig. S1A, top panel). The depression effect of DDX5 knockdown on p24 production in the presence of wild-type HIV-1 infection (Fig. 1F, top panel) is only 30%, which could be due to input of wild-type HIV-1 is relatively high and the knockdown efficiency of DDX5 by exogenous siRNA in primary human CD4+T cells is not good (only ∼27% decrease) (Fig. 1F, bottom panel). The same phenotype was recapitulated with another type of HIV-1 molecular clone (pNL4-3ΔEnv-GFP; Fig. S1A). Since several DEAD-box proteins (such as DDX1 and DDX3) are already known to be Rev co-factors, a substitution experiment was performed to detect the particular importance of DDX5. DDX1 or DDX3 overexpression after DDX5 knockdown only partially recued the function of DDX5 (Fig. S1B). All these results indicated that DDX5 is required for HIV-1 replication in human cells and has its particular importance to HIV-1 replication.

### DDX5 is Required for the Efficient Function of HIV-1 Rev

Previous studies showed that RNA helicase acted either transcriptionally or post-transcriptionally [33], [34]. As a member of the RNA helicase family, DDX5 might affect either Tat or Rev function. To clarify these possibilities, TZM-bl cells were co-transfected with a DDX5-expressing plasmid (or *DDX5*-specific siRNA) and HIV-1 Tat proteins. Neither DDX5-overexpression nor DDX5-knockdown affected the expression of the LTR luciferase reporter gene in TZM-bl cells (data not shown), indicating that DDX5 does not affect the function of Tat.

**Figure 2:**
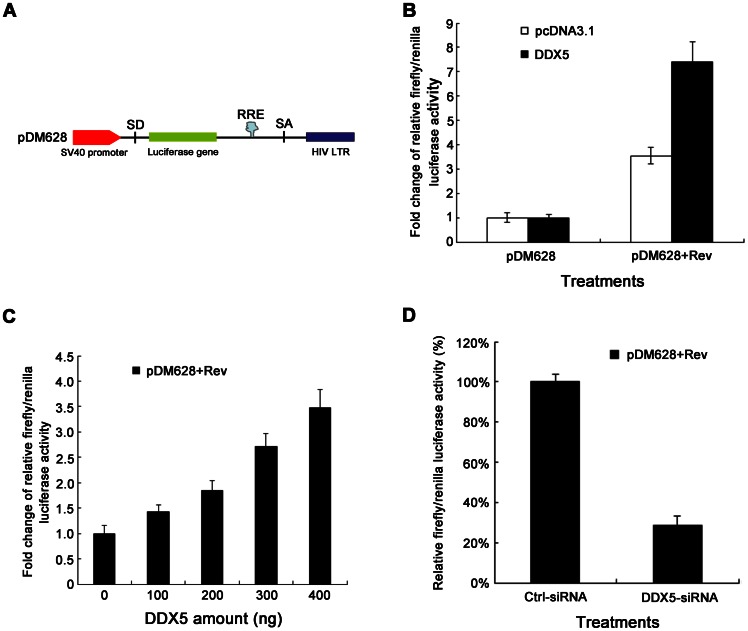
DDX5 significantly enhances the expression of a Rev-dependent reporter gene. **A**. Schematic diagram of construction of pDM628. **B** and **C**. The 293T cells were co-transfected with pDM628, pRL-TK (as a transfection normalization reporter), pcDNA3.1-Rev and pcDNA3.1-DDX5-HA (or pcDNA3.1). The cells were lysed at 48 h p.t. for luciferase activity assay. **D**. pDM628, pRL-TK, pcDNA3.1-Rev and DDX5-siRNA were transfected into 293T cells. After 48 h, cells were lysed for luciferase activity assay. Data in **B**, **C** and **D** represent mean ±S.D. (error bars).

Next, the Rev-responsive RRE-containing pDM628 plasmid was used to detect the effect of DDX5 on post-transcriptional gene regulation (Fig. 2A) [35]. The effects of DDX5 on *firefly luciferase* gene (expressed by pDM628) and *renilla luciferase* gene (expressed by pRL-TK, the transfection reporter) were detected. Fig. S2 showed that DDX5 did not influence the transcription from each reporter plasmids. DDX5 also did not affect the basal expression of pDM628 (15 ng) when DDX5 (60 ng) was expressed in 293T cells (Fig. 2B, lane1). However, when DDX5 (60 ng) was co-expressed with Rev (15 ng), the expression of the Rev-dependent reporter increased by ∼7-fold (Fig. 2B, lane 2). To further confirm this phenotype, a DDX5-dose-dependent experiment was performed. The enhancing effect of DDX5 on the reporter in pDM628 was found to correlate with the level of DDX5 expression (Fig. 2C). The 0 ng DDX5 transfection lane (lane 1 in Fig. 2C) still allows pDM628 expression possibly because of the expression of endogenous DDX5. After depletion of endogenous DDX5, we also recapitulated the similar phenotype (Fig. 2D). Collectively, these data suggested that DDX5 is important for the efficient function of HIV-1 Rev.

### DDX5 Regulates Rev/RRE- but not CTE-dependent Reporter Gene Expression

**Figure 3:**
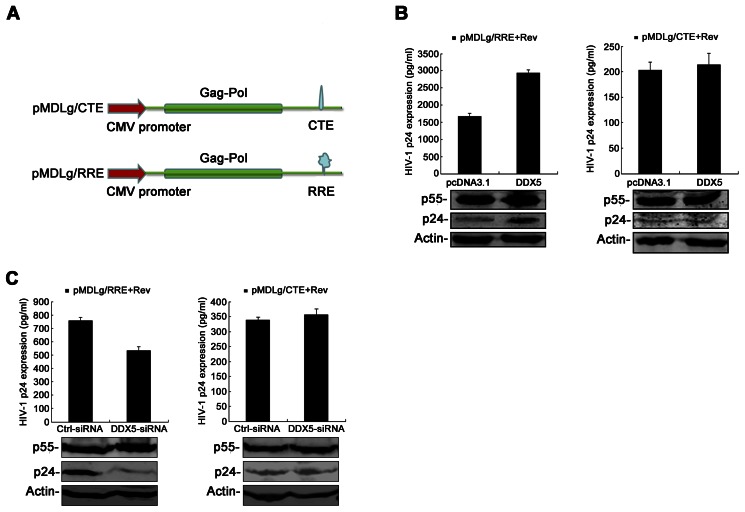
DDX5 modulates Rev/RRE- but not CTE-dependent reporter gene expression. **A**. Schematic maps of pMDLg/CTE and pMDLg/RRE. **B**. The 293T cells were co-transfected with pMDLg/RRE (or pMDLg/CTE), pcDNA3.1-Rev and pcDNA3.1-DDX5-HA (pcDNA3.1 as a control). **C**. The 293T cells were co-transfected with pMDLg/RRE (or pMDLg/CTE) and pcDNA3.1-Rev in the presence of DDX5-siRNA (GFP-siRNA as a control). **B** and **C**. The supernatants were collected for assay of HIV-1 p24 ELISA at 48 h p.t. Western blotting analysis was performed to confirm p55 Gag expression with anti-p24 antibody. The results are mean values with ±S.D. from triplicate samples.

As Rev is required for the export of HIV-1 Gag-encoding mRNA from the nucleus to cytoplasm by binding with an RRE sequence, the above results imply a role for DDX5 in an Rev/RRE-dependent shuttling function [3], [4], [6], [25]. To address this issue, pMDLg/RRE and pMDLg/CTE reporter plasmids were used to examine the effect of DDX5 on Rev/RRE-dependent export. Both of pMDLg/RRE and pMDLg/CTE are the CMV-driven Gag/Pol vectors. The pMDLg/RRE vector (Fig. 3A, bottom panel) contains the RRE element and expresses Gag protein in a Rev-dependent manner, whereas pMDLg/CTE (Fig. 3A, top panel) contains a MPMV constitutive transport element (CTE) and expresses protein in a Rev-independent manner [7]. Rev/RRE-dependent expression of p24 and p55 Gag was enhanced significantly by co-expression of DDX5 (Fig. 3B, left). However, when we repeated the above experiment with a Rev/RRE-independent pMDLg/CTE vector, the expression of p24 and p55 Gag was not affected by DDX5 co-expression (Fig. 3B, right). The same experiments were also performed in the presence of DDX5-siRNA (Fig. 3C) and consistent with the above results. Given that CTE-mediated expression of Gag was independent of Rev, pMDLg/CTE reveals traces of transport and p24 expression (Fig. 3B and C, right, bottom panel). All these results demonstrated that DDX5 functions specifically in Rev/RRE-dependent reporter gene expression.

### DDX5 Enhanced the Export of Rev/RRE-dependent mRNAs

**Figure 4:**
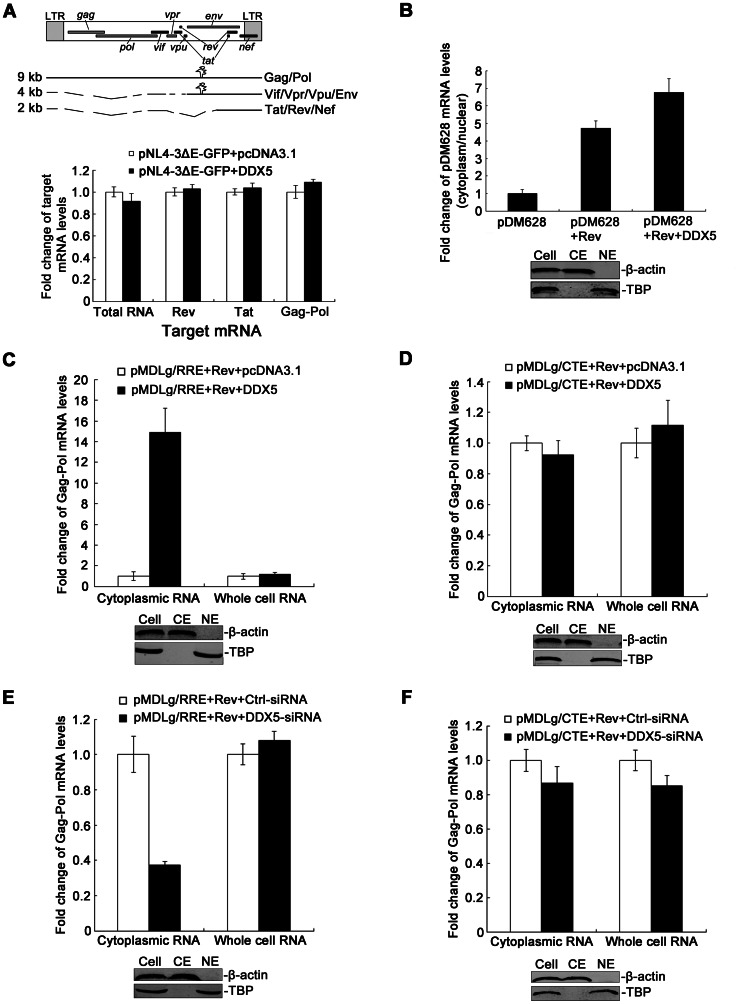
DDX5 enhanced the export of Rev/RRE-dependent mRNAs. **A**. Top, schematics of three size classes of HIV-1 RNAs. Bottom, the effect of DDX5 overexpression on HIV-1 mRNA splicing. The 293T cells were co-transfected with pNL4-3ΔEnv-GFP and pcDNA3.1-DDX5-HA (or pcDNA3.1), respectively. Total RNA was extracted from transfected cells and analyzed with qRT-PCR using primers specific to *rev* mRNA, *tat* mRNA or *gag-pol* mRNA. **B**. pDM628, pcDNA3.1-Rev and pcDNA3.1-DDX5-HA (pcDNA3.1 as a control) were transfected into 293T cells. Cells were collected for fractionation of cytoplasmic or nuclear components and then RNA extraction. Real-time RT-PCR was performed using primers specific to pDM628 mRNA. **C** and **D**. The 293T cells were co-transfected with pMDLg/RRE (or pMDLg/CTE), pcDNA3.1-Rev and pcDNA3.1-DDX5-HA (pcDNA3.1 as a control). **E** and **F**. The 293T cells were co-transfected with pMDLg/RRE (or pMDLg/CTE), pcDNA3.1-Rev (pcDNA3.1 as a control) and DDX5-siRNA (GFP-siRNA as a control). **C**, **D**, **E** and **F**. Cytoplasmic and whole-cell RNAs isolated from 293T cells at 48 h p.t. were analyzed using real-time RT-PCR. **B**, **C, D, E** and **F**. Human GAPDH and/or β-actin mRNA was measured as an endogenous control. The Western blotting images assess the purity of nuclear extracts (NE) and cytoplasmic extracts (CE): TBP measured as a NE control and β-actin measured as a CE control. Data in **A**, **B**, **C**, **D**, **E** and **F**. represent mean ± S.D. (error bars).

Since DDX5 functions as a co-factor of Rev and enhances the expression of the Rev-dependent reporter gene (Fig. 2 and Fig. 3), we then further exclude the possibility that this effect is not on transcription or splicing. We therefore co-transfected 293T cells with the HIV-1 molecular clones pNL4-ΔEnv-GFP and pcDNA3.1-DDX5-HA and analyzed HIV-1 RNAs by real time-PCR. HIV-1 mRNAs are divided into three size classes (Fig. 4A, top panel): The 9-kb unspliced RNA encodes Gag and Gag-Pol proteins; The 4-kb singly spliced mRNAs encode Vif, Vpu, Vpr, and Env; The 2-kb fully spliced mRNAs encode Tat, Rev, and Nef. The primer-binding sites for unspliced HIV-1 mRNAs were selected in the ORF (Open Reading Frame) of the *gag* gene, and the primer-binding sites for the fully spliced mRNAs were selected in the overlapping coding region of the *tat* and *rev* genes. Comparing the DDX5-expressing sample to controls, we observed no differences in the expression of total HIV-1 RNAs and spliced HIV-1 RNAs (Tat or Rev) (Fig. 4A, bottom panel). The same phenotype was recapitulated after DDX5 knockdown (Fig. S3). These results suggested that DDX5 cannot affect HIV-1 RNA transcription or splicing.

Next, we detected cytoplasmic or nuclear distribution of Rev-dependent pDM628 mRNA by DDX5 co-expression, and found that DDX5 co-expression exerted no significant effect on whole-cell pDM628 mRNA (data not shown). However, the cytoplasmic distribution of pDM628 mRNA increased significantly, indicating that DDX5 facilitates the export of Rev-dependent reporter mRNA from the nucleus to cytoplasm (Fig. 4B). As described above, we have shown that DDX5 regulates Rev/RRE-dependent but not CTE-dependent reporter gene expression. To determine whether DDX5 functions specifically in the export of Rev/RRE-dependent reporter gene mRNA, we performed the identical experiment with pMDLg/RRE (Fig. 4C) or pMDLg/CTE (Fig. 4D). Neither the whole-cell Gag-Pol RNA of pMDLg/RRE nor pMDLg/CTE was affected by DDX5 co-expression (Fig. 4C, right panel; Fig. 4D, right panel). Moreover, DDX5 did not affect the cytoplasmic distribution of Gag-Pol mRNA from pMDLg/CTE (Fig. 4D, left panel). However, the cytoplasmic distribution of Rev/RRE-dependent Gag-Pol mRNA was enhanced ∼13-fold by co-expression of DDX5 (Fig. 4C, left panel). After knockdown of endogenous DDX5, the cytoplasmic distribution of Gag-Pol mRNA from pMDLg/RRE reduced ∼60% (Fig. 4E, left panel) and the export of HIV-1 mRNA, which generated from pMDLg/CTE, did not change (Fig. 4F). These data suggested that DDX5 specifically facilitates the export of Rev/RRE-dependent reporter mRNA from the nucleus to cytoplasm.

### DDX5 Interacts with Rev in an RNA-dependent Way

**Figure 5:**
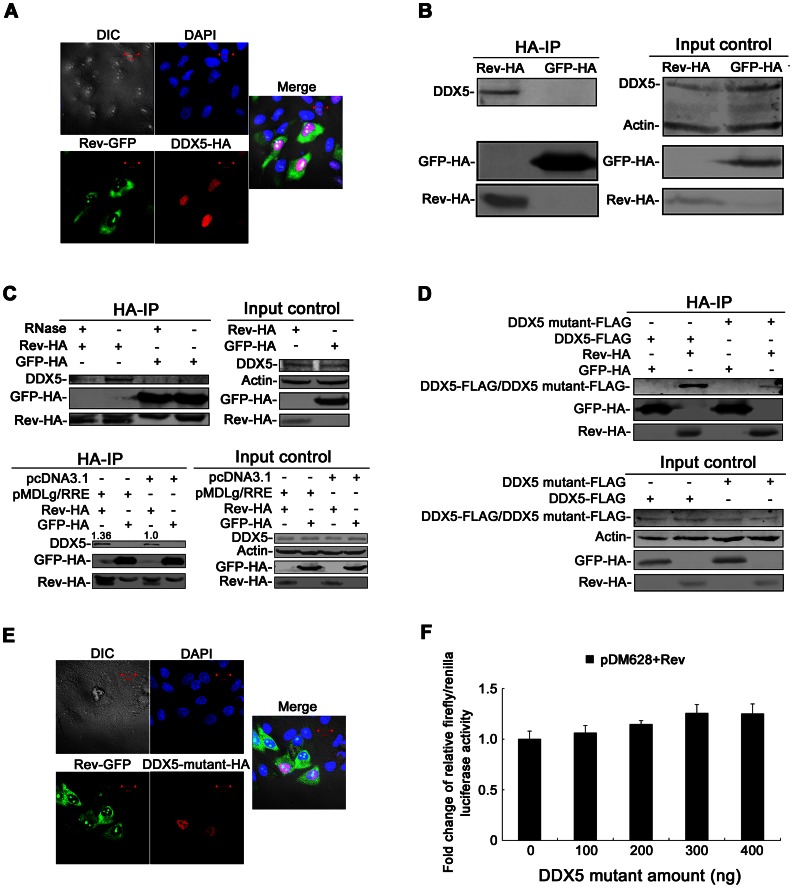
The interaction between DDX5 and Rev is largely dependent on RNA. **A** and **E**. Confocal images of transfected HeLa cells. **A**. HeLa cells were co-transfected with Rev-GFP- and DDX5-HA-expressing plasmids. **E**. HeLa cells were co-transfected with Rev-GFP- and DDX5 mutant-HA-expressing plasmids. **A** and **E**. At 36 h p.t., the transfected cells were observed for fluorescence by confocal laser scanning microscopy. **B** and **C** (Top panel). The transfected HeLa cells expressing Rev-HA or GFP-HA were lysed and immunoprecipitated with anti-HA agarose beads. **B**. Anti-HA immunoprecipitates were analyzed by Western blotting with anti-HA or anti-DDX5 antibody. **C** (Top panel). The immunoprecipitates were treated with or without RNase mixture. Samples were analyzed by immunoblotting using anti-HA or anti-DDX5 antibody. **C** (bottom panel). HeLa cells were co-transfected with pcDNA3.1-Rev-HA (pcDNA3.1-GFP-HA as a control) and pMDLg/RRE (pcDNA.3.1 as a control) were lysed and immunoprecipitated with anti-HA agarose beads. Anti-HA immunoprecipitates were analyzed by Western blotting with anti-HA or anti-DDX5 antibody. **D**. HeLa cells were co-transfected with pcDNA3.1-Rev-HA and pcDNA3.1-DDX5 mutant-FLAG (pcDNA3.1-GFP-HA and pcDNA3.1-DDX5-FLAG as controls). After immunoprecipitation with anti-HA antibody, the immunoprecipitated samples were analyzed with SDS-PAGE and immunoblotting with anti-HA or anti-FLAG antibody. **F**. The 293T cells were co-transfected with pDM628, pRL-TK (as a transfection normalization reporter), pcDNA3.1-Rev and differing amounts of pcDNA3.1-DDX5 mutant-FLAG (or pcDNA3.1). The cells were lysed at 48 h p.t. for luciferase activity assay.

As described above, DDX5 functions as a co-factor of Rev in HIV-1 replication. To further study the mechanism, the subcellular localization of DDX5 and Rev was first examined. For this purpose, 200 ng pEGFP-N1-Rev and 800 ng pcDNA3.1-DDX5-HA were co-transfected into HeLa cells. Cells were treated with immunofluorescence method and then examined with a confocal laser scanning microscope. The subcellular localization of Rev-GFP diffused throughout the cytoplasm and nucleus at 36 h p.t. (Fig. 5A). However, the localization of DDX5-HA was observed to congregate in the nucleus in transfected cells, which is consistent with a previous report (Fig. 5A) [24]. Thus, this result showed partially co-localization of Rev and DDX5, indicating that DDX5 binds to Rev in the nucleus.

To further study the interaction between DDX5 and Rev, HeLa cells were transfected with a Rev-HA- (6 μg) or GFP-HA-expressing plasmid (6 μg). After immunoprecipitation with anti-HA antibody, the immunoprecipitated samples were analyzed with SDS-PAGE and subsequently immunoblotted. As shown in Fig. 5B (left), DDX5 was specifically co-immunoprecipitated with Rev but not with the control GFP. Because RNA helicases usually interact with RNA and play a role in several RNA-related biological processes [12], [33], [34], the immunoprecipitated samples were then treated with RNase mixture. After RNase treatment, the interaction between DDX5 and Rev was reduced significantly and only a few DDX5 proteins were detected (Fig. 5C, top panel), indicating that the binding of DDX5 and Rev is largely dependent on some cellular RNAs. As HIV-1 transcript is also RNA, we hypothesized that the addition of HIV-1 transcript might affect the binding of Rev to DDX5. To confirm this speculation, 2 μg pMDLg/RRE (pcDNA3.1 as a control) and 4 μg pcDNA3.1-Rev-HA (pcDNA3.1-GFP-HA as a control) were co-transfected into HeLa cells and then these cell samples were immunoprecipitated with anti-HA antibody and analyzed by Western blotting (Fig. 5C, bottom panel). The data showed that HIV-1 transcript generated from pMDLg/RRE can enhance the interaction between Rev and DDX5.

As the DEAD-box motif plays an important role in the function of the DEAD-box RNA helicase family, the interaction between DDX5 with the DEAD-box mutant and Rev was evaluated. The pcDNA3.1-DDX5 mutant-FLAG or pcDNA3.1-DDX5 mutant-HA was generated by replacing the DEAD-box motif in DDX5 with 4 alanine amino acids. HeLa cells were then co-transfected with pcDNA3.1-Rev-HA and pcDNA3.1-DDX5 mutant-FLAG. The immunoprecipitated samples were analyzed with SDS-PAGE and immunoblotted after immunoprecipitation with anti-HA antibody. Compared with wild-type DDX5, the interaction between the DDX5-DEAD-box mutant and Rev was reduced significantly (Fig. 5D). After co-transfection with Rev, the localization pattern of DDX5 mutant was also detected. However, DDX5-DEAD-box mutant showed a diffuse nuclear staining but is largely excluded from the nucleoli (Fig. 5E). This mislocalization of DDX5 mutant maybe affect its ability to interact with Rev. Furthermore, the DDX5-DEAD-box mutant lacked the ability to enhance the expression of the Rev-dependent reporter in pDM628 (Fig. 5F). These results indicated that the DEAD-box motif of DDX5 is very important for the binding of DDX5 and Rev, and that it is also required for DDX5 to enhance Rev function.

## Discussion

Genome-wide screenings for HIV-1-associated cellular factors have recently been performed by several groups. Consolidating these data, more than 300 HIV-1-associated host factors have been found [5], [17], [18], [19], [20], [21], [22]. Bushman et al analyzed the full set of proteins implicated in HIV-1 infection and classified these factors into 11 clusters according to their putative functions. Of these clusters, the function of certain proteins in the eleventh cluster remains unknown, although they appear to be involved in a Rev-associated complex, suggesting specific link to HIV-1 replication [18]. DDX5 is among these cellular factors and our present works have demonstrated that DDX5 can enhance HIV-1 replication by interacting with Rev and positively affect its function.

DDX5, also known as p68 RNA helicase, is a member of the eleventh cluster and belongs to the DEAD-box RNA helicase family [18]. As an early example of cellular RNA helicases, DDX5 exhibits many functions, as do other RNA helicases; *e.g.,* the ATPase and RNA-unwinding activities of DDX5 have been previously identified [36], [37], [38]. DDX5 also plays a role in DNA methylation/demethylation, apoptosis, cell proliferation and early organ maturation [39], [40], [41]. In addition, several groups have suggested that DDX5 may be correlated with transcriptional regulation of a number of genes [39], [42], [43], [44], [45], [46]. Although DDX5, DDX1 and DDX3 belong to the DEAD-box RNA helicase family, DDX5 is quite different from DDX1 and DDX3 in either structure or function. For instance, DDX5 does not have a SPRY domain as DDX1 has, and the modification of its amino acids is much more significant than that of DDX1. For gene transcriptional regulation, DDX5 is a co-regulator of some transcription factors including ESR1, p53, MYOD1 and RUNX2, but this effect is not dependent on its ATPase/helicase activity [39], [43], [45], [47], [48]. Conversely, DDX1 acts as a co-activator to enhance NF-κB-mediated transcriptional activation and the expression of cyclin CCND2 [49], [50]. However, DDX3 enhances the expression of the CDKN1A/WAF1 promoter in a SP1-dependent manner and down-regulates the expression of the E-cadherin promoter [51], [52], [53]. For pre-mRNA processing, DDX5 is directly involved in the alternative regulation of some pre-mRNA splicing [54]; DDX1 may also be involved in 3′-end cleavage and polyadenylation of pre-mRNA [55], whereas DDX3 does not seem to be directly involved in splicing and rather associates with spliced mRNAs in an exon junction complex-dependent manner [56]. For regulation of apoptosis, DDX5 plays a role in the p53/TP53-dependent apoptotic pathway [39], [57]; DDX3 acts as an anti-apoptotic protein by association with GSK3 and cIAP-1 in a death-antagonizing signaling complex at death receptors to inhibit apoptotic signaling [58]. These significant functional differences show the diversity of the DEAD-box RNA helicase family and indicate that DDX5 has its own unique structure and function.

As we report here, DDX5 is shown to possess a novel function, acting as a co-factor of Rev; and is important for Rev/RRE-mediated HIV-1 mRNAs export. Our results showed that DDX5 overexpression enhanced HIV-1 replication significantly (Fig. 1A, E). To confirm this phenotype, a dose-dependence experiment was performed. We found that DDX5 enhanced HIV-1 p24 expression in a dose-dependent manner (Fig. 1C, D). Conversely, the depletion of DDX5 by *DDX5*-specific siRNA resulted in a significant inhibition of p24 production (Fig. 1F and Fig. S1A). These results are consistent with our own work (Fig. A–E) and previous studies which showed that the depletion of DDX1 or DDX3 reduced p24 expression significantly [9], [10]. Since DDX1 and DDX3 are already known to play a role in HIV-1 replication, a substitution experiment was performed to detect the particular importance of DDX5. The data in Fig. S1B showed that the expression of DDX1 or DDX3 in the presence of DDX5 knockdown only partially recued the function of DDX5, indicating that DDX5 has its unique function in HIV-1 replication.

However, our results disagree with the results in a recent report that DDX5 silencing strongly increased p24 release [59]. The authors of this report argued that DDX5 and DDX17 could form homodimers or heterodimers in cells [60], the silencing of DDX5 could increase the concentration of the DDX17 homodimers so as to enhance HIV-1 production. However, there is no evidence supporting their hypothesis. We used the same *DDX5*-specific siRNA as they used in their study (data not shown) and another *DDX5*-specific siRNA (Fig. 1F) to repeat this experiment. However, both of the results showed that the depletion of DDX5 reduced rather than increased p24 expression significantly.

A previous report has suggested that DDX5 and some cellular proteins are possibly involved in Rev-related function [18]. We therefore examined the relationship between DDX5 and the Rev-dependent reporter plasmid (pDM628, Fig. 2A) [9]. In line with the functions of DDX1 and DDX3, we found that DDX5 is required for the efficient function of Rev in a dose-dependent manner (Fig. 2B, C) and this phenotype is also recapitulated in the DDX5-silencing experiment (Fig. 2D). We also found that DDX5 regulates Rev/RRE- but not CTE-dependent reporter gene expression by using pMDLg/RRE and pMDLg/CTE reporter plasmids (Fig. 3). Zolotukhin et al showed that CTE-mediated expression of Gag is Rev independent [7]. In contrast to DDX5, another RNA helicase, RNA helicase A (RHA), plays a role in the nuclear export of CTE-containing RNAs [61]. RHA interacts with some shuttling proteins (TAP or a novel A kinase-anchoring protein HAP95) and displays its function by direct binding to CTE-containing RNA [62], [63], [64]. As neither the RHA/HAP95 nor the RHA/TAP complex affects the gene expression of Rev-RRE-dependent reporter [62], [64], and DDX5 does not affect the export of CTE-containing RNA, RHA and DDX5 belong to different RNA transport complexes and the nuclear export of CTE-containing RNAs or RRE-containing RNAs needs distinct co-factors.

As the main function of Rev is to bind with unspliced and partially spliced HIV-1 transcripts and shuttle them from the nucleus to the cytoplasm, DDX5 might participate in this activity as a co-factor binding with RNA and affecting either splicing or export of HIV-1 transcripts. Our results indicate that DDX5 augments the cytoplasmic accumulation (Fig. 4C) and cytoplasm/nuclear ratios of Rev/RRE-dependent mRNAs (Fig. 4B), but does not affect the splicing of HIV-1 RNAs (Fig. 4A and Fig. S3). To elucidate the mechanism underlying the phenotypes, we further detected the interaction between DDX5 and Rev by confocal microscopy and co-immunoprecipitation analysis. DDX5 was mainly found in the nucleus of transfected cells (Fig. 5A). This is consistent with a previous study that described DDX5 as a nuclear-cytoplasmic shuttling protein with a much longer residence time in the nucleus [24]. As Rev is a nuclear-cytoplasmic shuttling protein and some Rev proteins were also detected in the nucleus (Fig. 5A) and partially co-localized with DDX5 (Fig. 5A), our findings suggest that DDX5 partially co-localizes with Rev in the nucleus of human cells. Alternatively, our co-immunoprecipitation analysis further confirmed that DDX5 binds to Rev in an RNA-dependent manner and the interaction between DDX5 and Rev is promoted by some cellular RNAs or HIV-1 transcript (Fig. 5B, C). This is in partial agreement with previous reports regarding the interaction between DDX1 or DDX3 with Rev [9], [10]. As the DEAD-box motif is required for the function of DEAD-box RNA helicases, the localization pattern of DDX5-DEAD-box mutant and the binding of the DDX5 mutant and Rev were also examined. The co-immunoprecipitation and confocal imaging analysis indicated that the DEAD-box motif plays an important role in the interaction between DDX5 and Rev (Fig. 5D and E), and that it is also required for DDX5 to enhance Rev function (Fig. 5F).

Collectively, our analyses have identified that DDX5 functions as a new co-factor of Rev, facilitating Rev/RRE-mediated nuclear export of HIV-1 transcripts and enhancing the replication of HIV-1. Our data reveals a new potential molecular target for anti-HIV-1 therapeutics. As there are several active drug-research programs that target virus-encoded helicases [65], [66], it remains to be determined whether small molecular inhibitors upon the interaction between DDX5 and Rev can be found.

## Supporting Information

Figure S1 **DDX5 is important for HIV-1 replication.** **A**. The effect of DDX5 knockdown on HIV-1 p24 production. Top, the supernatants from 293T cells transfected with pNL4-3ΔEnv-GFP in the presence of DDX5-siRNA (GFP-siRNA as a control) were collected at 48 h p.t. and analyzed with p24 ELISA kit. Bottom, the effect of DDX5-siRNA in 293T cells was detected by Western blotting. **B**. Substitution experiment by DDX1 or DDX3. The 293T cells were transfected with pNL4-3ΔEnv-GFP and DDX5-siRNA (GFP-siRNA as a control). Then, DDX1- or DDX3-expressing plasmid was transfected into these cells. The culture supernatants were collected at 48 h p.t. for assay of p24 ELISA. Data in **A** and **B** represent mean ±S.D. (error bars).
(TIF)

Figure S2 **The effect of DDX5 on the transcription of reporter genes. A** and **B**. The 293T cells were co-transfected with pDM628 (**A**) or pRL-TK (**B**) and differing amounts of pcDNA3.1-DDX5-HA (pcDNA3.1 as a control), respectively. Total RNA was extracted from the transfected cells and analyzed with qRT-PCR using primers specific to *firefly luciferase* mRNA or *renilla luciferase* mRNA. Data in **A** and **B**. represent mean ± S.D. (error bars).
(TIF)

Figure S3 **The effect of DDX5 knockdown on HIV-1 mRNA splicing.** The 293T cells were co-transfected with pNL4-3ΔEnv-GFP and DDX5-siRNA (GFP-siRNA as a control), respectively. Total RNA was extracted from the transfected cells and analyzed with qRT-PCR using primers specific to *rev* mRNA, *tat* mRNA or *gag-pol* mRNA. Data represents mean ± S.D. (error bars).
(TIF)
