# Supplementary figures and images for: DDX5 Facilitates HIV-1 Replication as a Cellular Co-Factor of Rev
Source: PLoS One. 2013 May 31;8(5):e65040. doi: 10.1371/journal.pone.0065040 (PMC3669200; doi:10.1371/journal.pone.0065040)

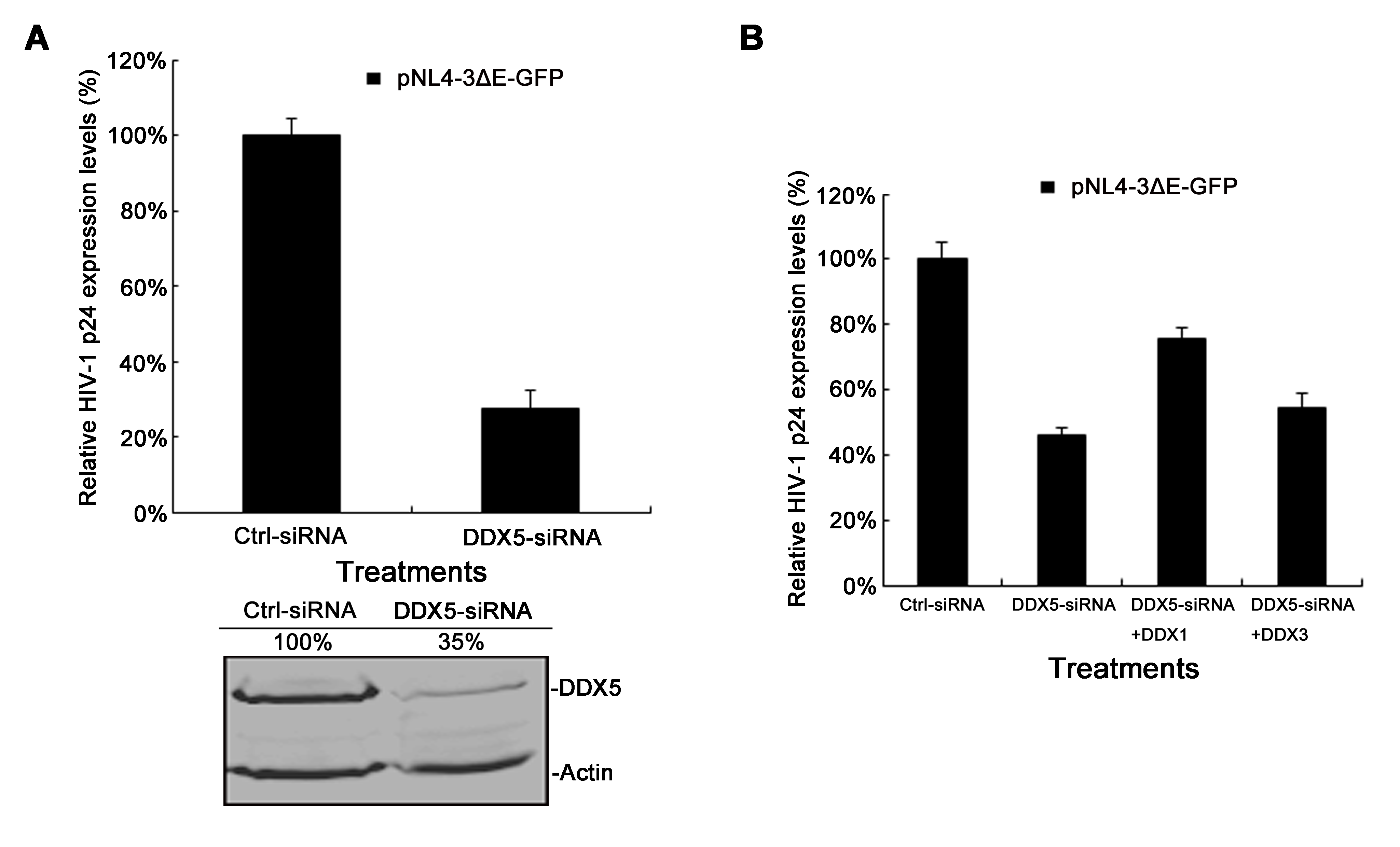

Supplement: Figure S1 — DDX5 is important for HIV-1 replication. A. The effect of DDX5 knockdown on HIV-1 p24 production. Top, the supernatants from 293T cells transfected with pNL4-3ΔEnv-GFP in the presence of DDX5-siRNA (GFP-siRNA as a control) were collected at 48 h p.t. and analyzed with p24 ELISA kit. Bottom, the effect of DDX5-siRNA in 293T cells was detected by Western blotting. B. Substitution experiment by DDX1 or DDX3. The 293T cells were transfected with pNL4-3ΔEnv-GFP and DDX5-siRNA (GFP-siRNA as a control). Then, DDX1- or DDX3-expressing plasmid was transfected into these cells. The culture supernatants were collected at 48 h p.t. for assay of p24 ELISA. Data in A and B represent mean ±S.D. (error bars). (TIF) [file pone.0065040.s001.tif]

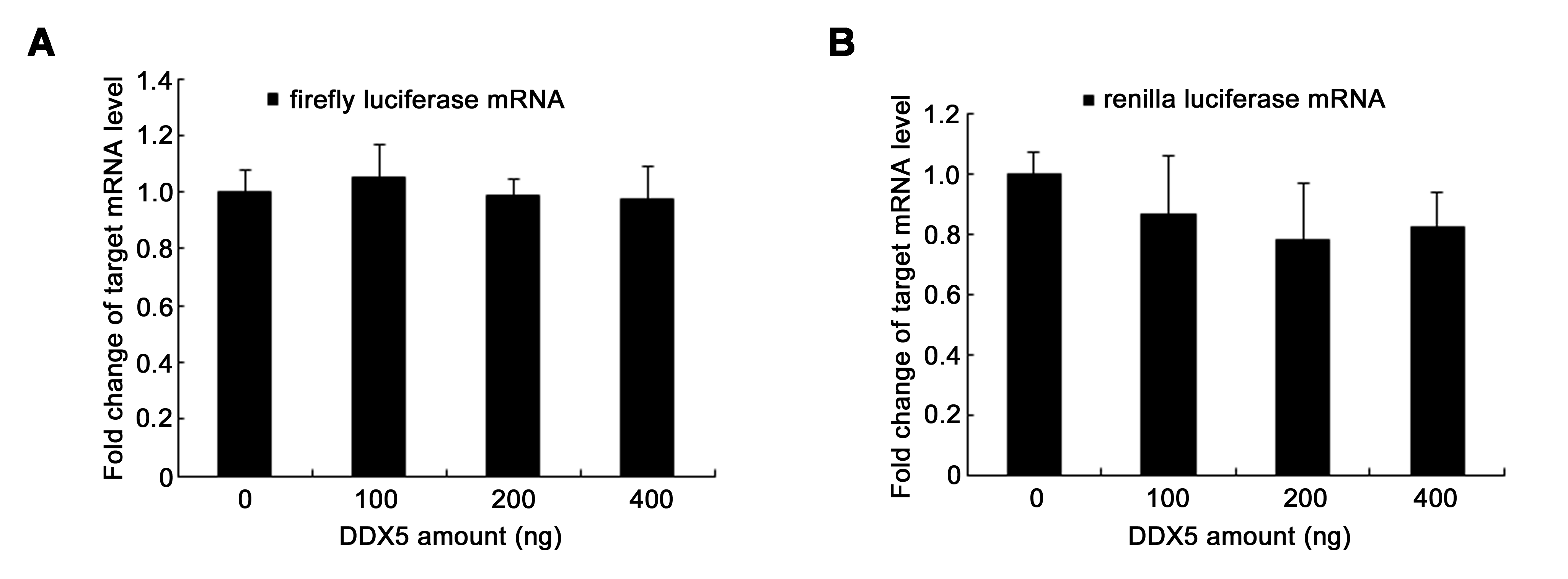

Supplement: Figure S2 — The effect of DDX5 on the transcription of reporter genes. A and B. The 293T cells were co-transfected with pDM628 (A) or pRL-TK (B) and differing amounts of pcDNA3.1-DDX5-HA (pcDNA3.1 as a control), respectively. Total RNA was extracted from the transfected cells and analyzed with qRT-PCR using primers specific to firefly luciferase mRNA or renilla luciferase mRNA. Data in A and B. represent mean ± S.D. (error bars). (TIF) [file pone.0065040.s002.tif]

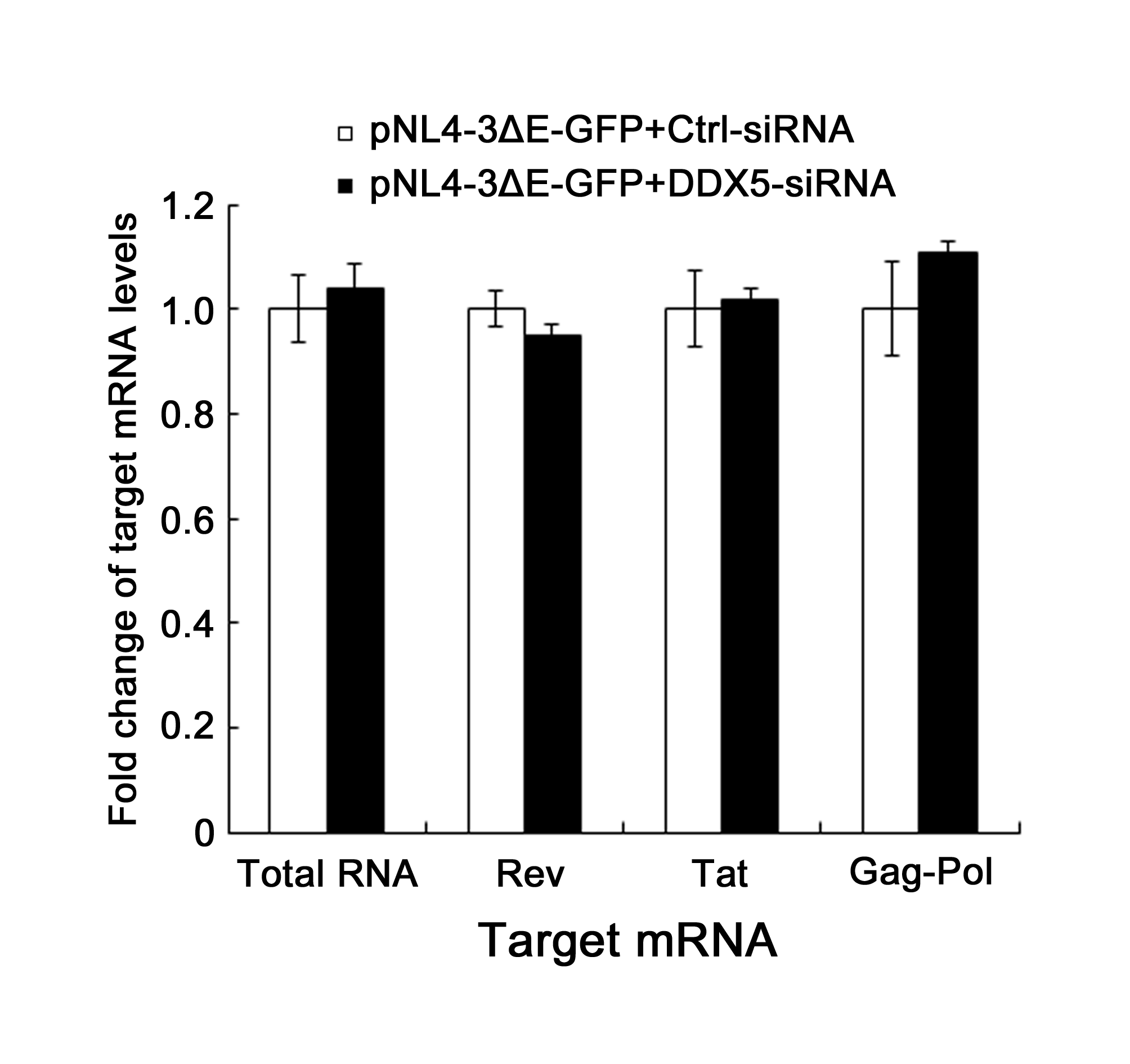

Supplement: Figure S3 — The effect of DDX5 knockdown on HIV-1 mRNA splicing. The 293T cells were co-transfected with pNL4-3ΔEnv-GFP and DDX5-siRNA (GFP-siRNA as a control), respectively. Total RNA was extracted from the transfected cells and analyzed with qRT-PCR using primers specific to rev mRNA, tat mRNA or gag-pol mRNA. Data represents mean ± S.D. (error bars). (TIF) [file pone.0065040.s003.tif]
